# Neurodevelopment and early pharmacological interventions in Fragile X Syndrome

**DOI:** 10.3389/fnins.2023.1213410

**Published:** 2023-08-02

**Authors:** Luis A. Milla, Lucia Corral, Jhanpool Rivera, Nolberto Zuñiga, Gabriela Pino, Alexia Nunez-Parra, Christian A. Cea-Del Rio

**Affiliations:** ^1^Centro de Investigacion Biomedica y Aplicada (CIBAP), Escuela de Medicina, Facultad de Ciencias Medicas, Universidad de Santiago de Chile, Santiago, Chile; ^2^Laboratorio de Neurofisiopatologia, Centro de Investigacion Biomedica y Aplicada (CIBAP), Escuela de Medicina, Facultad de Ciencias Medicas, Universidad de Santiago de Chile, Santiago, Chile; ^3^Physiology Laboratory, Department of Biology, Faculty of Science, Universidad de Chile, Santiago, Chile; ^4^Cell Physiology Center, Universidad de Chile, Santiago, Chile

**Keywords:** neurodevelopment, Fragile X syndrome, clinical trials, pharmacological interventions, GABA, mGluR, PI3K

## Abstract

Fragile X Syndrome (FXS) is a neurodevelopmental disorder and the leading monogenic cause of autism and intellectual disability. For years, several efforts have been made to develop an effective therapeutic approach to phenotypically rescue patients from the disorder, with some even advancing to late phases of clinical trials. Unfortunately, none of these attempts have completely succeeded, bringing urgency to further expand and refocus research on FXS therapeutics. FXS arises at early stages of postnatal development due to the mutation and transcriptional silencing of the Fragile X Messenger Ribonucleoprotein 1 gene (*FMR1*) and consequent loss of the Fragile X Messenger Ribonucleoprotein (FMRP) expression. Importantly, FMRP expression is critical for the normal adult nervous system function, particularly during specific windows of embryogenic and early postnatal development. Cellular proliferation, migration, morphology, axonal guidance, synapse formation, and in general, neuronal network establishment and maturation are abnormally regulated in FXS, underlying the cognitive and behavioral phenotypes of the disorder. In this review, we highlight the relevance of therapeutically intervening during critical time points of development, such as early postnatal periods in infants and young children and discuss past and current clinical trials in FXS and their potential to specifically target those periods. We also discuss potential benefits, limitations, and disadvantages of these pharmacological tools based on preclinical and clinical research.

## Introduction

Brain development occurs in a highly coordinated fashion, with a wide range of molecular and environmental factors contributing to neuronal network morphology, connectivity, and functional maturation. Fragile X Syndrome (FXS) is the leading genetic cause of intellectual disability and autism and is classified as a neurodevelopmental disorder due to the appearance of behavioral and cognitive phenotypes during early postnatal development. The syndrome exhibits a range of behavioral and cognitive alterations, including abnormalities in social behaviors, increased anxiety, cognitive deficits, hyperexcitability, and sensory hyper-responsiveness (Miller et al., 1999; Kau et al., 2004; Kaufmann et al., 2004; Hagerman and Stafstrom, 2009).

FXS is caused by the transcriptional silencing of the Fragile X Messenger Ribonucleoprotein 1 (*FMR1*) gene due to the hypermethylation of a CGG repeat expansion in its 5’-untranslated region (5’UTR; (Oberlé et al., 1991; Verkerk et al., 1991; Vincent et al., 1991; Yu et al., 1991). This epigenetic control mechanism is developmentally regulated (Mor-Shaked and Eiges, 2018) and occurs during early stages of gestation in different cells and to varying degrees (Schultz et al., 2015), even within the same tissue (Chen et al., 2011). The silencing of *FMR1* results in the loss of expression of the Fragile X Messenger Ribonucleoprotein (FMRP), an RNA-binding protein that primarily regulates both mRNA mobilization and local translation (Feng et al., 1997; Brown et al., 2001; Stefani et al., 2004), but it has been also involve in mRNA stability (De Rubeis and Bagni, 2010; Shu et al., 2020), DNA damage response (Alpatov et al., 2014), and direct ion channel regulation (Kalmbach and Brager, 2020; Kshatri et al., 2020). FMRP mRNA targets are involved in processes that occur during critical periods of postnatal neurodevelopment such as axonal guidance, synaptic connectivity, and neuronal network plasticity (Till, 2010) strongly suggesting that therapeutic intervention during these time windows could be relevant for completely or partially reversing the molecular and cellular alterations underlying the physiological symptoms observed in individuals with FXS.

In this review, we briefly discuss the role of FMRP during postnatal neurodevelopment, and then, based on age-group recruitment requirements, summarize the results of current and past pharmacological clinical trials in FXS. Finally, we discuss the potential benefits, limitations, and disadvantages of early postnatal pharmacological interventions.

## Role of FMRP in neurodevelopment

Since FMRP is a RNA-binding protein that regulates protein translation by association with mRNAs, its absence impacts different neurodevelopmental processes along this time frame (Russo and DiAntonio, 2019; Sears et al., 2019; Doll et al., 2020, 2021; Prashad and Gopal, 2021). In FXS, FMRP is progressively developmentally regulated with epigenetic silencing of the *FMR1* gene that begins around 10 to 13 weeks of gestation (Willemsen et al., 2002; Li et al., 2020), and cognitive and behavioral FXS characteristics emerging during early childhood (2- to 3-year-old).

To study the role of FMRP during brain development, researchers have widely used rodents as an experimental model where the *Fmr1* gene is knocked-out from zygote stages in the FXS mice model (The Dutch-Belgian Fragile X Consorthium et al., 1994). Evidence from rodents developmental brain suggest that the first postnatal week is roughly equivalent to the third semester human infant (Clancy et al., 2007; Semple et al., 2013), including processes such as oligodendrocyte maturation (postnatal day (pnd) 1-3) and increased axonal and dendritic density (pnd 7-10) (Semple et al., 2013). However, there are still some processes that occur postnatally in both humans and rodents such as peaks in synaptogenesis, peak in myelination rate, neurotransmitter receptor changes (humans: 2-3 year old; rodents: pnd 20-21), and plateau for synaptic pruning (humans: 12–18 year old; rodents: pnd 35-49) (Semple et al., 2013; Silbereis et al., 2016). In the mouse model, FMRP brain expression peaks seem to coincide with critical periods of synaptogenesis (Zito and Svoboda, 2002), which occurs at the end of the first postnatal week (Bonaccorso et al., 2015), and gradually declines thereafter (Nimchinsky et al., 2001; Gholizadeh et al., 2015). Recent studies have shown that re-expression of FMRP in cortical excitatory cells during early postnatal development ameliorates structural, functional, and behavioral abnormalities seen in the FXS mouse model (Rais et al., 2022).

**Figure 1:**
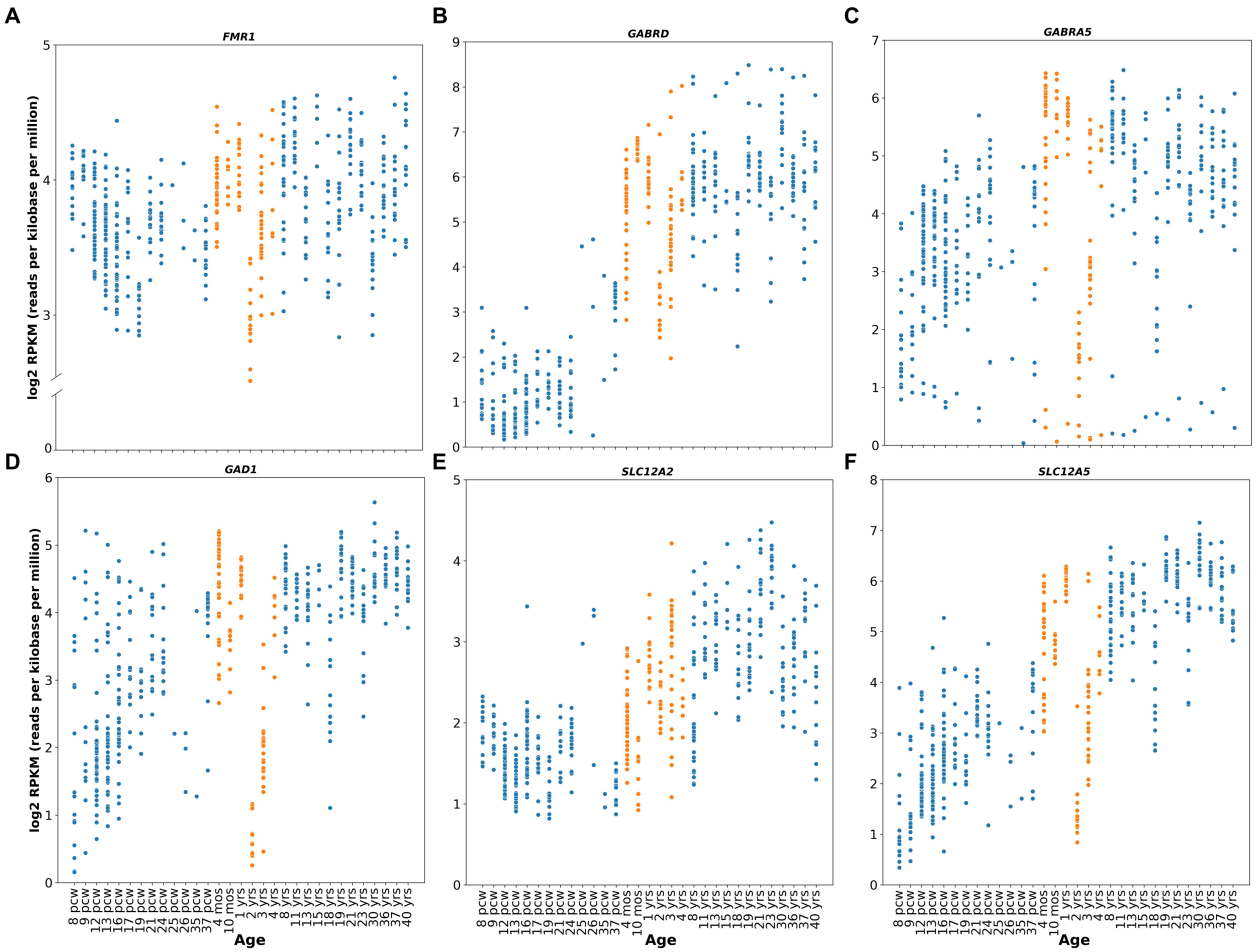
Human relative gene expression throughout development. FMR1, GABRD, GABRA5, GAD1, SLC12A2 and SLC12A5 genes from embryonic stages (post conceptional weeks, pcw) to adulthood of healthy human subjects. RNA-seq data were obtained from Brainspan Developmental Transcriptome, containing log2 RPKM (reads per kilobase per million) values. Orange dots shows expression from 4 months to 4 years. Genes analyzed were: **(A)** FMR1 (ENSG00000102081), **(B)** GABRD (ENSG00000187730), **(C)** GABRA5 (ENSG00000186297), **(D)** GAD1 (ENSG00000128683), **(E)** SLC12A2 (ENSG00000064651), **(F)** SLC12A5 (ENSG00000124140). RNA-seq data were obtained from Brainspan Developmental Transcriptome, publicly available at https://human.brain-map.org/.

FMRP expression also seems to peak perinatally in healthy human subjects, as suggested by the relative expression levels of the gene (Figure 1A) obtained from the publicly available Brainspan – Allen Brain Atlas website (Miller et al., 2014). This correlates with neurodevelopmental milestones such as synaptogenesis, synaptic pruning, myelination and apoptosis (Silbereis et al., 2016). Furthermore, this role of FMRP during neurodevelopment is highlighted by one of the most characteristic neuronal phenotypes found in FXS: an abundance of hyper elongated and immature dendritic spines observed in both animal models (Zito and Svoboda, 2002; Kang et al., 2021) and human cortical brain regions (Irwin et al., 2000, 2001). Additionally, studies from forebrain organoids from patient-derived induced pluripotent stem cells (iPSC) reveal that loss of FMRP leads to dysregulated neurogenesis, abnormal neuronal differentiation, increased neuronal excitability and pervasive gene expression alterations in a cell-specific manner (Sunamura et al., 2018; Kang et al., 2021). More specifically, iPSC from FXS patients showed a delayed GABA polarization switch, decrease number of GABAergic inhibitory interneuron populations and hyperexcitable membrane properties in differentiated neurons (Zhang et al., 2022). These findings correlate with diminished GABA, GABA receptors, and glutamate decarboxylase (GAD) expression levels, the delayed GABA polarization switch, and the overall neuronal hyperactivity reported in animal models (Cea-Del Rio et al., 2014; Contractor et al., 2015; Di et al., 2020).

Altogether, evidence suggests that FMRP plays an important function during neurodevelopment, particularly at the synaptogenesis peri- and postnatal critical periods, which may underlie the clinical symptomatology observed in the mature FXS nervous system, providing an attractive window for therapeutic intervention.

## Current and potential use of small molecules during neurodevelopment for FXS

**Figure 2:**
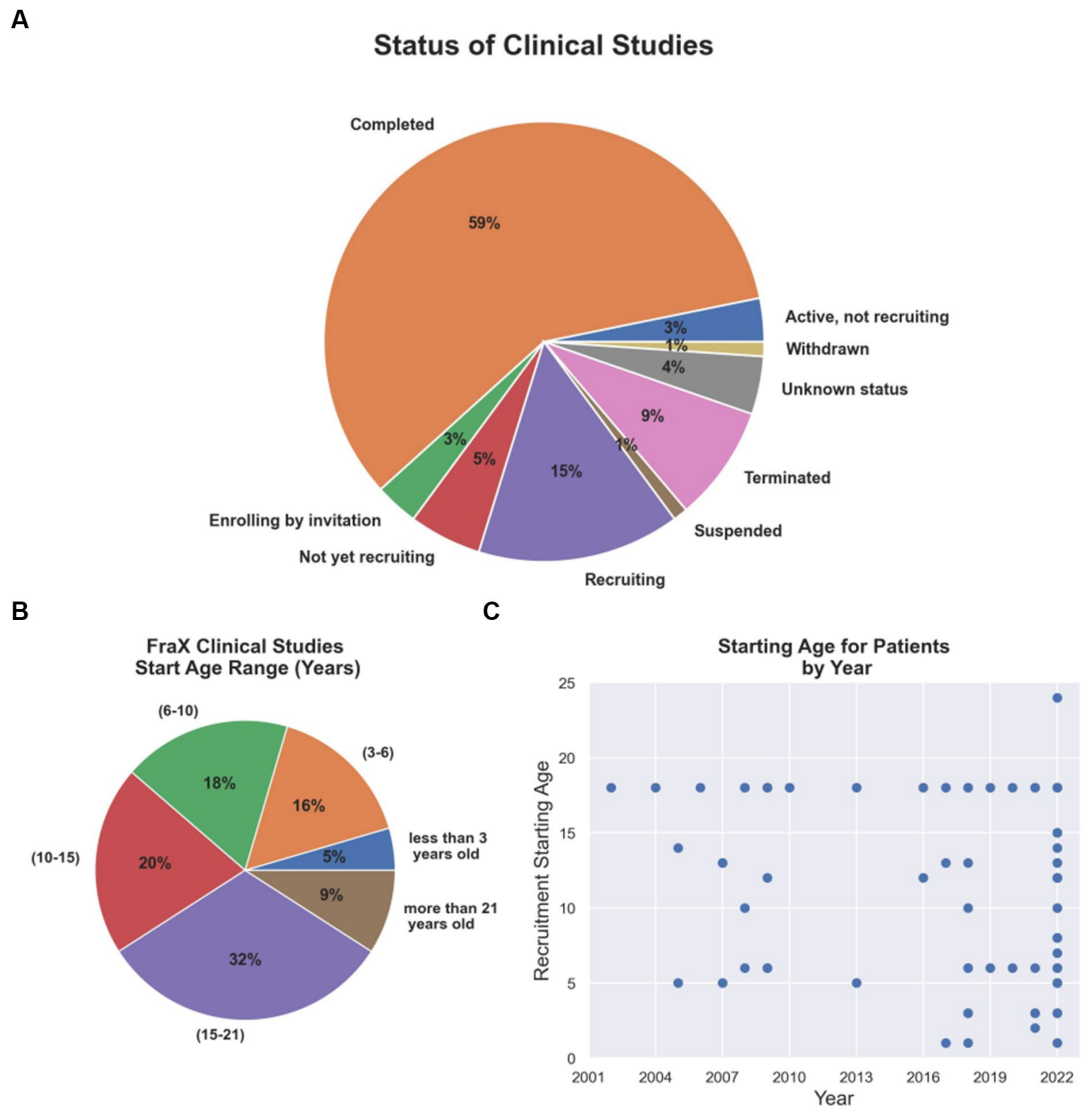
Current FXS clinical trial status and age group addressed. **(A)** Completion and recruiting status for FXS clinical trials. **(B)** Percentage of FXS clinical trials per age group. **(C)** Starting age required for patients recruited in clinical trials by year from 2002 to 2022.

Since 2002, several therapeutic tools and approaches have been tested in clinical trials for FXS (Supplementary Table S1; The data included in this table was obtained on August 2022 from www.ClinicalTrials.gov; search terms *“Fragile X Syndrome,” “FXS.”* The table is available in the following repository https://doi.org/10.6084/m9.figshare.23643210.v1). Although the results have mostly been unfavorable because reason such as outcome measures chosen, enrollment criteria fidelity, enhanced placebo response rates and what age range is most appropriate to treatment success, they have helped to redefine or concentrate efforts on newer and multidisciplinary approaches (Reviewed in Erickson et al., 2017). Thus, clinical trials have begun to focus on addressing early postnatal temporal windows that may coincide with critical periods of neurodevelopment (Figures 2B,C). To this day, only 5 and 16% of the FXS clinical trials have focused on populations younger than 3 years old or between 3 to 6 years old children, respectively (Figure 2B), although numbers have started to trend upward in recent years (Figure 2C).

Pharmacological approaches have concentrated on drug design, addressing mGluR antagonism, GABA modulation, and intracellular pathway modulation (Berry-Kravis et al., 2017; Ligsay et al., 2017; Hagerman et al., 2018; Youssef et al., 2018). The details of the pharmacological agents tested and results from preclinical studies and clinical trials have been widely and recently reviewed (Erickson et al., 2017; Munshi et al., 2017; Berry-Kravis et al., 2018; Protic et al., 2022), so we will focus on discussing literature that highlights the potential pharmacological use during critical time windows of neurodevelopment and the possible outcomes.

### Metabotropic glutamate receptor neurotransmission and signaling

Synaptic activation regulates FMRP expression via mGluRs (Weiler et al., 1997) that rely on intracellular pathways associated with mTOR and ERK1/2 signaling (Nosyreva and Huber, 2006; Sharma et al., 2010), a pathway that is particularly relevant during neurodevelopmental periods of synaptogenesis. These findings form the basis of the most prominent theory explaining FXS, the “mGluR theory,” which involves the upregulation of mGluR-dependent mechanisms and increased protein synthesis (Bear et al., 2004). This theory has been validated in preclinical studies in mice and *Drosophila melanogaster* models where several FXS cellular and behavioral phenotypes were corrected when mGluR neurotransmission was either antagonized or knocked out (Chuang, 2005; McBride et al., 2005; Dölen et al., 2007).

Over the years, different clinical trials have targeted the metabotropic glutamatergic hypothesis, including the testing of Mavoglurant, a selective antagonist of the mGlu5 receptor (ClinicalTrials.gov Identifiers: NCT01253629, NCT01433354), and Basimglurant (ClinicalTrials.gov Identifier: NCT01517698, NCT01015430, NCT01750957) and Fenobam (Berry-Kravis et al., 2009), both negative allosteric modulators of the same receptor. Fenobam was discontinued after a pilot study that did not report results due to financial problems with the manufacturer company, and Basimglurant’s trial status has not been updated since 2016. Mavoglurant has reported a lack of efficacy for behavioral phenotypes in several clinical trial studies, regardless of FMRP methylation status when evaluated in adolescent/adult groups (Berry-Kravis et al., 2016).

Interestingly, despite being a neurodevelopmental disorder, clinical trials have only recently begun to focus on exploring pharmacological interventions during early developmental periods (Figure 2B). Indeed, a double-blind, placebo-controlled research design for Mavoglurant paired with an intensive language intervention is currently ongoing in children between 32 months and 6 years old (ClinicalTrials.gov Identifier: NCT02920892) assessing early postnatal windows of neurodevelopment that may overlap with periods of synaptogenesis and synaptic plasticity. The expected outcomes of this trial are to determine whether the drug improves language learning and communication that involve the child’s use of gestures, eye contact, vocalizations, and/or words and word combinations to communicate a message to a listener (ClinicalTrials.gov Identifier: NCT02920892).

This pursuit of an early postnatal intervention is supported by evidence showing that group 1 mGluRs (mGluR1 and mGluR5) have an important role in synaptogenesis, synaptic pruning, and plasticity (Riedel and Reymann, 1996; Bortolotto et al., 1999, 2005; Cea-Del Rio et al., 2020), which occur during perinatal and early postnatal development. Indeed, mGluR5 protein expression levels are high during the first 2 weeks of postnatal life (Lu et al., 1997; Friedman et al., 2016; Lum et al., 2016) and studies in mGluR5 KO mice have shown impaired learning behavior and defects in the formation of the somatosensory cortical barrels, underscoring its importance for cortical development (Catania et al., 1994; Romano et al., 2002). Moreover, downregulation of mGluR expression (Dölen et al., 2007) or administration of mGluR antagonists to neonate *Fmr1* KO mice (Su et al., 2011) had a greater effect on reducing the average neuronal spine length and density of the FXS phenotype when compared to adults. On the contrary, mGluR antagonism during early stages of wild type mice development, although at higher concentrations to those reported in *Fmr1* KO, have also shown diminished proliferation and differentiation of progenitor neuronal cells (PNCs) (Di Giorgi-Gerevini et al., 2005; Friedman et al., 2016), similar to those exhibited by mice lacking mGluR5 (Di Giorgi-Gerevini et al., 2005). Furthermore, a study in FXS forebrain organoids from human showed that MPEP treatment failed to rescue the synaptic, excitability, and neuronal developmental phenotypes seen in these organoids (Kang et al., 2021), altogether, suggesting none or potential negative effect of mGluR antagonist drugs used during these windows of development.

In addition, considerations need to be made in light of recent human studies using positron emission tomography (PET) after administration of a novel specific mGluR5 PET ligand, 3-[^18^F]fluoro-5-(2-pyridinylethynyl) benzonitrile ([^18^F]FPEB), which showed reduced mGluR5 availability and distribution in humans with FXS (Brašić et al., 2021, 2022; Mody et al., 2021) either reflecting mGluR5 high occupancy, as a result of a hyperexcited network, or mGluR decreased protein expression. Interestingly, this last scenario would not be consistent with the proposed mGluR theory and the preclinical studies that gave rise to the clinical trials on mGluR antagonists (Huber et al., 2002; Bear et al., 2004; Osterweil et al., 2010). It is worth notice, that these PET studies were performed in FXS adult patients between 18 and 60 years old, which may explain the unsuccessful results in mGluR5 antagonist clinical trials that have addressed a similar age range (Bailey et al., 2015; Berry-Kravis et al., 2016; Youssef et al., 2018). Although this does not necessarily mean that pharmacological interventions during early development (children and infants) would be unsuccessful, a complex scenario for trials in younger patients could be expected. Beyond these considerations, further evaluation of mGluR5 expression in younger FXS patients is required to better understand the implications of the use of mGluR antagonists at these developmental stages.

### Excitatory-inhibitory balance and GABAergic neurotransmission

Excitatory-inhibitory (E-I) balance establishment depends on genetic and homeostatic mechanisms while experiences shape it throughout synaptogenesis and plasticity development (Sohal and Rubenstein, 2019). Indeed, E-I balance is crucial for maintaining stable levels of activity and as a signal-to-noise detector mechanism in the neuronal network (Sohal and Rubenstein, 2019). FXS is characterized by a hyperexcitable neuronal phenotype that primarily reflects disturbances on the E-I balance and its molecular components (Contractor et al., 2015; Antoine et al., 2019). Data form FXS animal models indicate that FMRP interacts with and regulates several of these components including NMDA, AMPA, GABA_A_ receptor subunits (GABA_A_R; The Dutch-Belgian Fragile X Consorthium et al., 1994; Gross et al., 2011; Deng and Klyachko, 2016; Zhu et al., 2018) and ion channel expression (Bureau et al., 2008; Harlow et al., 2010; Deng et al., 2013; Bausch et al., 2015). For instance, FMRP interacts with potassium channel subunits (Deng et al., 2013), and large-conductance BK calcium-activated channels (Bureau et al., 2008; Harlow et al., 2010) which regulate cell excitability. During neurodevelopment these alterations in the E-I balance have been shown to translate into delayed cortical functional maturation and disrupted synaptic plasticity in FXS (Olmos-Serrano et al., 2010; Cellot and Cherubini, 2013). Clinically, this hyperexcitable phenotype is proposed as a neurobiological substrate for behavioral traits such as anxiety, irritability, hyperactivity, and hypersensitivity, which explains why it has been considered a primary target in several clinical trials attempting to modulate either or both excitatory and inhibitory neurotransmission (ClinicalTrials.gov Identifiers: NCT00054730, NCT00895752, NCT00584948, NCT01911455, NCT03697161, NCT01725152, NCT01282268, NCT01325220, NCT03697161, and NCT01911455).

In particular, GABA_A_R was first postulated as a therapeutic target for the treatment of FXS in 2007 (D’Hulst and Kooy, 2007). These attempts were supported for several studies showing that *Fmr1*KO mice exhibit diminished levels of the GAD enzyme expression (Semple et al., 2013), and concomitant alterations in intracellular GABA availability and a reduction in synaptically released GABA (Olmos-Serrano et al., 2010). Also, FMRP interacts with GABA_A_R subunits (D’Hulst et al., 2006; Gantois et al., 2006; Curia et al., 2009; Olmos-Serrano et al., 2010; Kratovac and Corbin, 2013) that are typically associated with providing non-synaptic tonic forms of inhibition (Gantois et al., 2006; Curia et al., 2009; Martin et al., 2014; Zhang et al., 2017), a mechanism that delivers a persistent inhibitory background conductance that directly regulates the E-I balance (Mitchell and Silver, 2003; Semyanov et al., 2004; Bonin et al., 2007). These preclinical findings have been validated in humans, which show reduced GABA_A_R binding, as evaluated using PET scan (D’Hulst et al., 2015) and transcranial magnetic stimulation (TMS), revealing abnormal functional inhibition in adults with FXS (Morin-Parent et al., 2019). Subsequent preclinical studies with the GABA-mimetic Gaboxadol, a GABA_A_R agonist with higher affinity for delta(δ)-containing GABA_A_R (Stórustovu and Ebert, 2006), Ganaxolone, an allosteric GABA_A_R superagonist, and Arbaclofen, a GABA_B_R agonist, demonstrated correction of the locomotor hyperactivity, irritability, anxiety-like behaviors (Olmos-Serrano and Corbin, 2011; Cogram et al., 2019), audiogenic seizures (Heulens et al., 2012), repetitive/preservative behavior (Braat et al., 2015), and excessive basal protein synthesis and abnormal spine density (Pacey et al., 2009; Henderson et al., 2012; Qin et al., 2015) phenotypes.

In humans, clinical studies of Arbaclofen and Ganaxolone were undertaken in 2013 and 2016, respectively (ClinicalTrials.gov, NCT01725152, NCT01282268 and NCT01325220), with results reported in 2017 (Berry-Kravis et al., 2017; Ligsay et al., 2017). Unfortunately, neither of these drugs have resulted in significant improvements in the overall population, although promising results were observed when children population subgroups were separately analyzed (ages 6-9 years for Ganaxolone, and ages 5-11 years for arbaclofen) (Berry-Kravis et al., 2017; Ligsay et al., 2017). Specifically, Ganaxolone produced an improvement in stereotypic behaviors such as anxiety and cognitive abilities, and Arbaclofen showed specificity for irritability behaviors, which are relevant to social avoidance in human FXS. Berry-Kravis and collaborators argue that this effect could be explained because of the higher doses given to these groups compared to the adult group, or because these patients had higher levels of baseline irritability, which makes it easier to observe a positive response to the drug (Berry-Kravis et al., 2017). However, these results might also be explained by the age at which they received treatment. This last argument is especially relevant considering that the role of GABA in early development includes migration stimulation and guidance and the sculpting of the glutamatergic synaptic network (Owens and Kriegstein, 2002; Cellot and Cherubini, 2013), potentially correcting alterations in neurodevelopment. In this scenario, other potential GABA agents that could be tested in preclinical studies include modulators of tonic inhibition, such as selective agonists for alpha5 and delta subunits, which have relative gene expression peak levels (GABRD and GABRA5) at early postnatal stages in healthy human subjects (Figures 1B,C; https://human.brain-map.org/; Miller et al., 2014), but appear to be downregulated during neurodevelopment in FXS patients (Bonin et al., 2007; Martin et al., 2014). A new double-blind, placebo-controlled phase 2 clinical trials for Gaboxadol, which targets tonic inhibition specifically, is currently ongoing (ClinicalTrials.gov Identifier: NCT03697161). However, this study only includes adolescent and adult subjects (ages 13 to 22 years), limiting the possibility of assessing the potential for early postnatal therapeutic interventions in the GABAergic system.

Moreover, studies addressing early developmental stages using GABA-mimetic drugs should consider that proper maturation of the GABAergic system is critical to the E-I balance. Interventions during these periods could either promote or hinder these processes. For instance, in the immature mouse brain (P0 - P5) GABA acts to depolarize most neurons, due to the chloride (Cl^−^) electrochemical driving force which promotes an inward Cl^−^ conductance on activation of GABA receptors. This phenomenon is dominated by the developmental expression of 2 Cl^−^ co-transporter proteins, NKCC1 (SLC12A1) and KCC2 (SLC12A5). The differential developmental projection of their relative expression results in a switch of the Cl^−^ electrochemical gradient during the first postnatal week in mice (He et al., 2014) determining whether activation of the GABA_A_R is depolarizing or hyperpolarizing (Rivera et al., 1999). In the FXS mouse model, the normal progression of the GABA_A_R-mediated polarity switch is delayed, occurring during the second postnatal week instead (He et al., 2014), a delay also seen in differentiated iPSC from FXS patients (Zhang et al., 2022). In this context, preclinical studies addressed this alteration using Bumetanide and Furosemide to inhibit the chloride co-transporter NKCC1 during early postnatal development. Thus, both compounds were able to rectify the disrupted driving force through GABA_A_Rs in cortical neurons, restoring their synaptic development and cortical circuit function in FXS mice (He et al., 2019). Considering that these drugs are already FDA-approved to treat other disorders, and that the establishment of new indications for existing drugs has been proposed as an efficient alternative over the *de novo* drug development (a concept known as drug repurposing), these are not only new pharmacological treatment options but also support the hypothesis of early developmental interventions.

Studies also show that GABA itself can regulate the GABA polarity switch by modulating the expression of the KCC2 transporter (Ganguly et al., 2001; Heubl et al., 2017; Wright et al., 2017). This suggests that the decrease in GABA neurotransmission observed in *Fmr1* KO may explain the observation of delayed dynamics and temporal polarity switch of the GABA_A_R-mediated inhibitory responses in these animals. This will then impact the E-I balance of the network generating neuronal hyperexcitability as is seen in FXS. From here, although it may seem paradoxical to suggest the use of GABA mimetic drugs at early developmental stages since they may cause further excitability on the already hyperexcitable network in FXS, it can be speculated that administration of GABA-mimetic drugs early in postnatal development may have a beneficial effect by upregulating the KCC2 co-transporter. Upregulating KCC2 would promote the reversal of the Cl-electrochemical gradient and the mature hyperpolarizing GABA response. Such a manipulation may act to re-establish the temporality of the GABA switch and in turn rescue or restore the E/I balance and ameliorate the hyperexcitable phenotype. Despite the above, currently it is not known when this developmental switch occurs in humans. However, some evidence indicates that the expression of these transporters is developmentally regulated (Figures 1E,F; https://human.brain-map.org/; Miller et al., 2014). High NKCC1 and negligible levels of KCC2 protein are seen during perinatal stages, and gradual downregulation of NKCC1 and upregulation of KCC2 take place after the early postnatal period (Dzhala et al., 2005; Jansen et al., 2010; Sedmak et al., 2016), further suggesting a potential temporal window for postnatal pharmacological GABA-mimetic intervention.

### Intracellular pathways

The FXS cellular phenotype also relies in faulty functioning of intracellular pathways including hyperactivation of mitogen-activated protein kinase (MAPK) and extracellular signal-regulated kinases (ERK) (Soong et al., 2008; Weng et al., 2008; Wang et al., 2012), as well as low levels of cAMP (Berry-Kravis et al., 1995; Wang et al., 2012; Gurney et al., 2017). These pathways are essential in several neurodevelopmental processes, including the transition from pluripotent stem cells to neuronal progenitors and synaptic plasticity, greatly impacting the cortical cytoarchitecture, organization and function (Iroegbu et al., 2021).

Clinical trials have tested different drugs targeting the restoration or correction of these pathways including: BPN14770, a Phosphodiesterase 4D (PDE4D) allosteric inhibitor (Clinical Trial Identifier: NCT03569631); Lovastatin, a FDA-approved specific inhibitor of the rate-limiting enzyme in cholesterol biosynthesis, 3-hydroxy-3-methylglutaryl coenzymeA [3HMG-CoA] reductase (Clinical Trial Identifier: NCT02680379 and NCT02642653); and Metformin, an insulin-normalizing drug that downregulates the insulin/IGF-I signaling pathway (Clinical Trial Identifier: NCT03862950 and NCT03479476). BPN14770 has only been tested in adult subjects (18-to 41-year-old) meeting the primary outcome measure for tolerability and the secondary outcome for cognitive performance and language in phase 2 clinical trials (Berry-Kravis et al., 2021). Importantly, PDE4D has been implicated in cognitive ability with the observations that (i) missense mutations of PDE4D cause rare neurodevelopmental disorder with intellectual disabilities (Acrodysostosis type 2) and (ii) PDE4D plays a role in regulating levels of cAMP which is an important neurobiological substrate of learning and memory early in development. Thus, future studies looking at the effects of BPN14770 at earlier ages are warranted to further explore potential benefits of an early pharmacological intervention.

On the other hand, both Metformin and Lovastatin clinical trials have addressed early time periods of development including younger children of 6, 8 and 10-year-old (Clinical Trial Identifier: NCT02680379, NCT02642653, NCT03862950 and NCT03479476). These studies showed a decrease in the aberrant behavior checklist total score (ABC) in the case of Metformin (Proteau-Lemieux et al., 2021), and significant improvements in ABC-community global score (ABC-C) when Lovastatin is combined with Minocycline, an antibiotic that inhibit matrix metallopeptidase 9 (MMP-9) showing potency in correct dendritic spine abnormalities in the *Fmr1* KO mouse (Champigny et al., 2022). Moreover, when Lovastatin was combined with parent-implemented language intervention (PILI), language and communication skills were improved (Thurman et al., 2020) supporting the benefits of interventions in early development. These treatments downregulate the phosphatidylinositol-3 kinase (PI3K) serine–threonine-specific protein kinase (AKT1) pathway, which is hyperactivated in animal models of FXS (Pellerin et al., 2016; Gantois et al., 2019; Altable and de la Serna, 2021). Furthermore, preclinical studies showed that inhibition of PI3K signaling normalizes the abnormal protein synthesis and altered neurogenic cell fate during development in FXS organoids (Kang et al., 2021; Raj et al., 2021). These results highlight again the therapeutic potential for intervention in early stages of development in humans, although in this case addressing embryonic periods would add complexity to pharmacologically intervene. Finally, a recently published controlled trial with Metformin showed improvements in memory, social novelty deficits, and neuroanatomical abnormalities in nine young children with FXS (2- to 7-year-old) (Biag et al., 2019). This has led to a new clinical trial for Metformin in children from 2 to 16-year-old with FXS to assess treatment of behavior, cognitive and language phenotypes (ClinicalTrial Identifier: NCT05120505).

Finally, it is worth to mention that some newer pharmacological intervention attempts, discussed in detail in other recent review articles (McBride et al., 2016; Berry-Kravis et al., 2018; Tartaglia et al., 2019; Protic et al., 2022), have tested compounds acting upon monoaminergic, oxytocinergic and endocannabinoid neurotransmission systems, including sertraline, oxyctocin, a cannabidiol transdermal gel (ZYN002), sulindac and methylphenidate, among others (ClinicalTrial Identifiers: NCT01474746, NCT01254045, NCT04823052, NCT03614663, NCT04977986 and NCT05301361). Most of these newer alternatives have made further efforts in addressing earlier time points in development, recruiting toddlers and preschoolers from as early as 3 or 6-year-old (Figure 2). Only two clinical trials have reported results in this population: sertraline which showed no benefit to the outcomes proposed (Potter et al., 2019), and oxytocin which reported some promising effects on social anxiety (Hall et al., 2012).

## Conclusion

More efforts need to be done to address the weaknesses and pitfalls of translating preclinical results from animal models studies to clinical research in order to properly identify and confirm the potential benefits of pharmacological treatments during critical periods of neurodevelopment. The *Fmr1* KO mice and *fmr1 Drosophila* models have allowed important progress in the FXS field but it is unclear how their molecular, cellular, and physiological features, as well as timeline or trajectories of development, translate to humans. For instance, in the FXS mice the *Fmr1* gene is knocked out resulting in the loss of FMRP expression since the zygote stage as opposed to the gradual decline in gene expression observed in humans. This limits the translation of developmental studies in FXS mice to humans. *Drosophila melanogaster* has been extensively used to study genetic and molecular aspects of human disease by implementing state-of-the-art technologies. Still, limitations arise since the results necessarily need to be further tested in mammalian-derived neurons, to better translate this information to physiological analyzes in complete tissues and finally to escalate to complex behavior studies. Currently, utilization of FXS iPSC-derived neurons and human brain organoid models are particularly informative since they both retain the epigenetic memory and exhibit a methylated FMR1 gene. Moreover, iPSCs can potentially differentiate into all cell types in large numbers providing a powerful platform for drug screening. This is particularly relevant for FXS research, where human neurons are inaccessible for studies other than from aborted fetuses or postmortem brain samples. There are, however, limitations with these experimental models in regard that they usually represent early fetal brain development stages, but it is unclear how they may shed light on optimal postnatal neurodevelopmental time windows for human pharmacological interventions in FXS. Fortunately, a recent study has shown that cortical organoids from neurotypical humans could mature to parallel *in vivo* postnatal development and maintain developmental milestones for 250 to 300 days postnatal (Gordon et al., 2021), further validating its value for late-onset associated critical neurodevelopmental periods and drug screening.

Finally, there are several barriers in performing clinical trials in a younger population, including physiological heterogeneity within the pediatric population (which can be divided at least in four different categories), scarce knowledge on pharmacokinetics and pharmacodynamics, the need for higher safety standards, the potential short and long-term negative side effects on the developing brain, and regarding legal consent for participation and balancing risk and benefits for this pediatric population. However, considering that FXS is a neurodevelopmental disorder, a new venue for interventions targeting relevant neurodevelopment time windows associated with the FXS phenotypes need to be more deeply considered. Although this strategy is already utilized in several clinical trials that have included 2-and 3-year-old children, further preclinical investigation is required to better understand at what age time point and how long therapeutic intervention should be given, in addition to knowing the potential positive and negative outcomes of such a treatment. Information from these studies could provide better strategies on how to avoid unwanted side effects, improve FXS phenotypes, and overall, how to improve the lifestyle of patients.

## Author contributions

CC-D developed the idea and topic of the manuscript. CC-D, LM, and AN-P contributed to the ideas expressed in the manuscript. LM design and create figures. LC, JR, NZ, and GP contributed to collecting, analyzing, and selecting the literature included in this manuscript. LC and JR write an original draft. CC-D, LM, and AN-P write, review, and edit the final version of the manuscript. All authors contributed to the article and approved the submitted version.

## Funding

This work is supported by Universidad de Santiago de Chile, Vicerrectoría de Investigación, Desarrollo e Innovación, DICYT 022001CDR to CC-D and DICYT 021801MB to LM.

## Conflict of interest

The authors declare that the research was conducted in the absence of any commercial or financial relationships that could be construed as a potential conflict of interest.

## Publisher’s note

All claims expressed in this article are solely those of the authors and do not necessarily represent those of their affiliated organizations, or those of the publisher, the editors and the reviewers. Any product that may be evaluated in this article, or claim that may be made by its manufacturer, is not guaranteed or endorsed by the publisher.

## Supplementary material

Supplementary Table S1 Pharmacological interventions in Clinical FXS trials since 2002. Dta included in this table was obtained on August 2022 from www.ClinicalTrials.gov; search terms “Fragile X Syndrome,” “FXS.”
